# Early Determinants of Adverse Motor Outcomes in Preschool Children with a Critical Congenital Heart Defect

**DOI:** 10.3390/jcm11185464

**Published:** 2022-09-16

**Authors:** Maaike C. A. Sprong, Barbara C. H. Huijgen, Linda S. de Vries, Hanna Talacua, Kim van Loon, Rian M. J. C. Eijsermans, Joppe Nijman, Johannes M. P. J. Breur, Marco van Brussel, Martijn G. Slieker

**Affiliations:** 1 Center of Child Development, Exercise and Physical Literacy, Wilhelmina Children’s Hospital, University Medical Center Utrecht, KG 02.056.0, P.O. Box 85090, 3508 AB Utrecht, The Netherlands; 2 Department of Psychology, Faculty of Behavioral and Social Sciences, University of Groningen, 9712 TS Groningen, The Netherlands; 3 Department of Neonatology, Wilhelmina Children’s Hospital, University Medical Center Utrecht, 3508 AB Utrecht, The Netherlands; 4 Department of Pediatric Cardiology, Wilhelmina Children’s Hospital, University Medical Center Utrecht, 3508 AB Utrecht, The Netherlands; 5 Division of Anaesthesiology, Intensive Care and Emergency Medicine, University Medical Center Utrecht, 3508 AB Utrecht, The Netherlands; 6 Pediatric Intensive Care Unit, Wilhelmina Children’s Hospital, University Medical Center Utrecht, 3508 AB Utrecht, The Netherlands

**Keywords:** critical congenital heart disease, children, preschoolers, cardiac surgery, neurodevelopmental outcomes, risk factors, motor development, Bayley-III-NL, Movement ABC-II-NL

## Abstract

Neurodevelopmental disabilities are common in infants with critical congenital heart disease (CCHD). A prospective, longitudinal cohort study was conducted to establish the prevalence and early determinants of adverse motor outcomes in infants who underwent cardiac surgery with cardiopulmonary bypass before six months of age. Motor development was assessed in 147 preschoolers using the Movement Assessment Battery for children-II. Although the majority displayed an average motor development, 22% of preschool children with CCHD deteriorated in their motor developmental score compared to their previous assessment at 18 months, especially in those with an aortic arch anomaly (AAA) (35%). Individual stability over time appeared to be moderate and the number of children with a motor delay increased, up to 20% in children with AAA. Motor development up to 42 months was best predicted by gestational age, cardio pulmonary bypass time, aortic cross clamp time, number of heart catheterizations up to 18 months and early motor outcomes. The increase in number of preschool children with a motor delay underlines the importance of longitudinal screening of motor skills in children with CCHD at risk for adverse motor outcomes. Offering early interventions may protect their current and future cardiovascular health as motor development is an independent predictor of exercise capacity, physical activity and participation in daily living.

## 1. Introduction

Congenital heart disease (CHD) occurs in approximately 9 per 1000 live births and corresponds to 1.35 million newborns worldwide every year [1]. CHDs are the leading cause of infant death from birth defects [2,3]. Around one-fourth of all CHDs require early diagnosis and surgical correction in the first months of life, e.g., hypoplastic left-heart syndrome, transposition of the great arteries and tetralogy of Fallot, and are therefore considered critical [2]. The mortality rate of infants with critical congenital heart disease (CCHD) has markedly declined over recent decades due to major advances in cardiac surgery and perioperative care [4]. Neurodevelopmental disabilities are common in infants with CCHD who have undergone open-heart surgery with cardiopulmonary bypass. Specific pre-, peri- and postoperative factors such as brain development, age at surgery, cardio pulmonary bypass (CPB) time, number and duration of hospital stays and lower pre- and postoperative neurological scores can contribute to postoperative brain damage and long-term neurological impairments [5,6,7,8,9]. However, these factors are inconsistently associated with outcome [10]. The exact causal pathways of abnormal motor development appears to be multifactorial and are not fully understood [5]. Neurodevelopmental disabilities vary from mild and barely detectable to severe motor developmental delays [11]. These delays are concerning as they pose a potential risk for future limited physical and educational achievements, reduced social interaction and diminished quality of life [5]. With regular neurological evaluations, developmental disorders associated with CCHD can be identified early on. Identifying risk factors may help predict the likelihood and severity of future motor delays and can be used in informing and advising parents on early treatment options.

Longitudinal studies on motor development and risk factors for adverse motor outcomes at (pre)school age and beyond in children with specific types of CCHD are scarce and report contradictory results [9,12,13,14,15]. In a recent study from our research group, we found that the number of infants and toddlers with delayed gross motor development was increased and increases with age in all CCHD subtypes [16]. Additional research is needed to determine whether the number of children with a motor delay increases further at preschool age or whether they grow out of their initial developmental delay. Therefore, the primary aim of this study is to investigate the motor performance of preschool children with CCHD who underwent open-heart surgery before six months of age and to describe possible differences in motor performance between the different types of CCHD. As it is clinically important to adequately predict motor deficits to intervene early and prevent complications later in life, we also aim to identify risk factors that contribute to adverse developmental outcomes at 42 months.

## 2. Materials and Methods

### 2.1. Study Design

This prospective observational cohort study is part of the institutional developmental follow up program named “Hart op Weg” at the Wilhelmina Children’s Hospital, University Medical Center Utrecht, Utrecht University, Utrecht, the Netherlands. Children with CCHD who underwent cardiac surgery with cardiopulmonary bypass within the first six months of life between July 2011 and November 2021 were invited to participate in the developmental follow up as part of standard care.

Between 2011 and 2016, all patients with transposition of great arteries (TGA), tetralogy of Fallot (TOF) and single ventricle physiology (SVP) were invited to participate. From 2016 onwards, infants with other types of CCHD such as aortic arch Anomalies (AAA) (e.g., hypoplastic aortic arch, interrupted aortic arch, hypoplastic left heart complex), aortic stenosis and pulmonary stenosis and total anomalous pulmonary venous connection (TAPVC) were also invited for follow-up. According to the Ethics Committee of the University of Utrecht, this study was not subject to the Medical Research Involving Human Subjects Act (WMO) (reference number 13/442; 5 September 2013). Signed parental informed consent was obtained for participation in our registry.

### 2.2. Participants

During the study period, 440 infants with CCHD were eligible for enrollment in our developmental outpatient clinic. Exclusion criteria for current data analysis included confirmed genetic anomalies such as trisomy 21, 22q11 deletion or CHARGE syndrome and prematurity, defined as birth before 36 weeks of gestation, and infants born with a birth weight below the 5th percentile because these infants are at increased risk of developmental disorders regardless of their CHD [17,18].

### 2.3. Outcome Assessment

At 3, 9, 18 and 42 months, all children underwent neurodevelopmental examination by an experienced developmental pediatrician/neonatologist and a pediatric physical therapist experienced in developmental assessment. Neurological examination was performed at each follow-up visit, including cranial nerves (eye contact/tracking movements, facial motor function), muscle tone (overall, axial, limbs) and strength (arms/legs), reflexes and signs of asymmetry.

At 42 months, the movement assessment battery for children—second edition (Movement ABC-II-NL) was used to investigate the motor performance and identify children with motor problems. Dutch normative data were used. The Movement ABC-II-NL is a global test of motor competence, with assessments of both fine and gross motor coordination. This test is divided into three different age bands; (3–6, 7–10, and 11–16 years). Each age band consists of eight items which are divided into three subsets: manual dexterity, aiming and catching, and balance. Raw scores are converted into standard scores (1–19) and percentile scores (0–100). Scores above the 16th percentile are regarded as average motor performance. Scores between the 6th and 16th percentile are considered ‘at risk’ for motor difficulties and scores below the 6th percentile indicate significant motor difficulties. The Movement ABC-II-NL has reasonable to good clinical utility in identifying children with motor performance differences [19].

In order to analyze longitudinal motor development, motor outcomes measured with Bayley-III at the ages of 3, 9, and 18 months were used. The Bayley-III-NL was selected as it is a normed, validated, and reliable tool for assessing gross and fine motor development. Total motor composite scores (mean 100, SD 15) FM and GM scaled scores (mean 10, SD 3) were used. To determine individual stability of motor developmental outcomes over time and compare the results of the different assessment tools, percentile scores and z-scores were used. In both the Movement ABC-II-NL and the Bayley-III-NL, the motor developmental scores were categorized as ‘delayed’ when percentile scores were ≤ P5, ‘at risk for motor difficulties’ when > P5 but ≤ P16 and ‘average’ when percentile scores were above 16 [19].

### 2.4. Statistical Analysis

Continuous variables are presented as mean ± SD as normally distributed. Normality of distribution of continuous variables was tested with the Kolmogorov–Smirnov test and visualized by skewness and kurtosis of histograms. If not normally distributed, medians with interquartile range were reported. Categorical variables are presented as frequencies and percentages. Differences in parametric, non-parametric, and dichotomous outcomes were analyzed using ANOVA, Kruskal–Wallis or chi-square test. Risk factors that may be associated with adverse motor outcomes were selected and obtained from patient files. Factors associated with motor development at 42 months were examined using spearman’s rho, chi-square, and fisher’s exact tests. Statistical analyses were performed with IBM SPSS^®^ version 25.0 (SPSS, Chicago, IL). A *p*-value < 0.05 was considered significant.

To investigate longitudinal changes in motor development over time, multilevel modeling by means of a mixed effect model was applied with the multilevel program MLwiN 3.04 (Centre for Multilevel Modelling, University of Bristol, Bristol, United Kingdom) [20]. A 2-level multilevel structure was used; level 1 represented repeated measures (at 3, 9, 18 and 42 months) and level 2 represented the participants. Variables that correlated significantly (*p*-value of < 0.05) in the univariate analysis were included in the model because these variables were considered to predict and or influence motor development. These predictors were entered into the model to find the best model fit. The model fit was evaluated by comparing the deviance (ln −2 log-likelihood) of the empty model from the final model. The empty model is the null model (i.e., lacking predictors), and as predictors are added to the model, the deviance changes. Predictors were only included in the model if statistically significant and clinically meaningful (estimate > 0.001) A simpler model can be rejected with a decrease in deviance and a *p* value of less than 0.05. An alpha level of 0.05 was adopted for all tests of significance.

## 3. Results

### 3.1. Patient Characteristics

**Figure 1:**
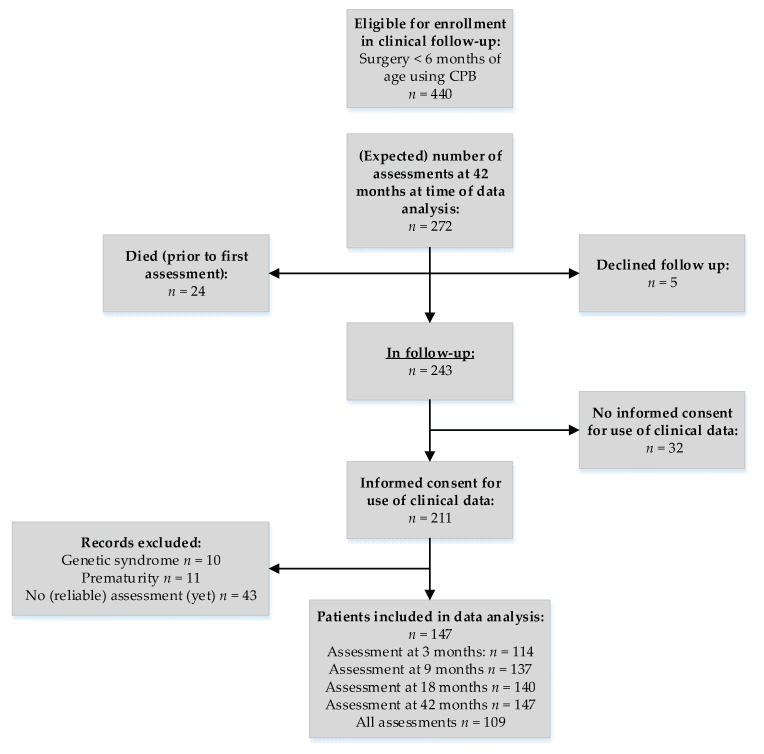
Flowchart enrollment in follow-up.

Of all the 440 children eligible for follow-up, 272 children met the inclusion criteria of the study. Of these 272 children, 24 infants (8.8%) died prior to the first examination at three months, and parents of 5 infants declined follow-up or did not respond. Of the 243 remaining children, parents of 32 children (13%) did not consent to the use of their clinical data. A total of 147 infants with a complete Movement ABC-II-NL score fulfilled the inclusion criteria. Details about patient enrollment are displayed in Figure 1.

Forty-three of the children who were initially in follow-up and had given informed consent to use their data had no assessment at 42 months. Reasons for no (complete) assessments were as follows: no show (*n* = 9), extensive care required elsewhere due to serious brain injury or comorbidity (*n* = 4), stopping follow-up at the request of parents, e.g., due to moving (*n* = 10), not yet scheduled due to planning delays as a result of the corona pandemic (*n* = 7). Finally, thirteen children could not be tested completely or reliably because they were insufficiently cooperative or task-oriented. Their assessment was therefore not included in the data analysis. The group of children who had no assessment at 42 months did not differ significantly from the included children in terms of age at surgery (31.7 days versus 25.7 days) (*p* = 0.59) diagnosis distribution (*p* = 0.516) and sex distribution (67% versus 63% boys) (*p* = 0.56). Patient characteristics, including CCHD diagnoses, are presented in Table 1. Detailed information on type of surgery is presented in Table A1.

### 3.2. Motor Development at 42 Months

One hundred forty-seven preschool children with a CCHD completed the motor assessment at a mean age of 43.3 (±1.97) months. The mean motor development score, assessed with the Movement ABC-II-NL, was 9.5 ± 3.14. A total motor developmental score ≤ P5 was seen in 13 (8.8%) children. Nine (6.1%), 13 (8.8%), and 8 (5.4%) children showed a motor delay (≤P5) in the subdomains manual dexterity, aiming and catching and balance, respectively. Detailed information about the distribution of total motor classifications at 42 months is displayed in Table 2.

### 3.3. Motor Development per Diagnosis Group

When the results of the different diagnosis groups were analyzed separately, there was a significant difference in total motor outcome between diagnosis groups at all ages (*p* < 0.05). Children with SVP and AAA scored significantly lower than children with TGA. Differences in motor outcome between SVP and AAA and the other diagnosis groups were not significant at 42 months. The percentage of children with an improved motor classification (12% was highest in the SVP group, the percentage of children with a deterioration in motor classification was highest in the AAA group (35.7%). Detailed information per diagnosis group about the longitudinal motor development and the distribution of total motor classifications is displayed in Table 2.

### 3.4. Motor Outcomes at 42 Months Related to Early Motor Development

**Figure 2:**
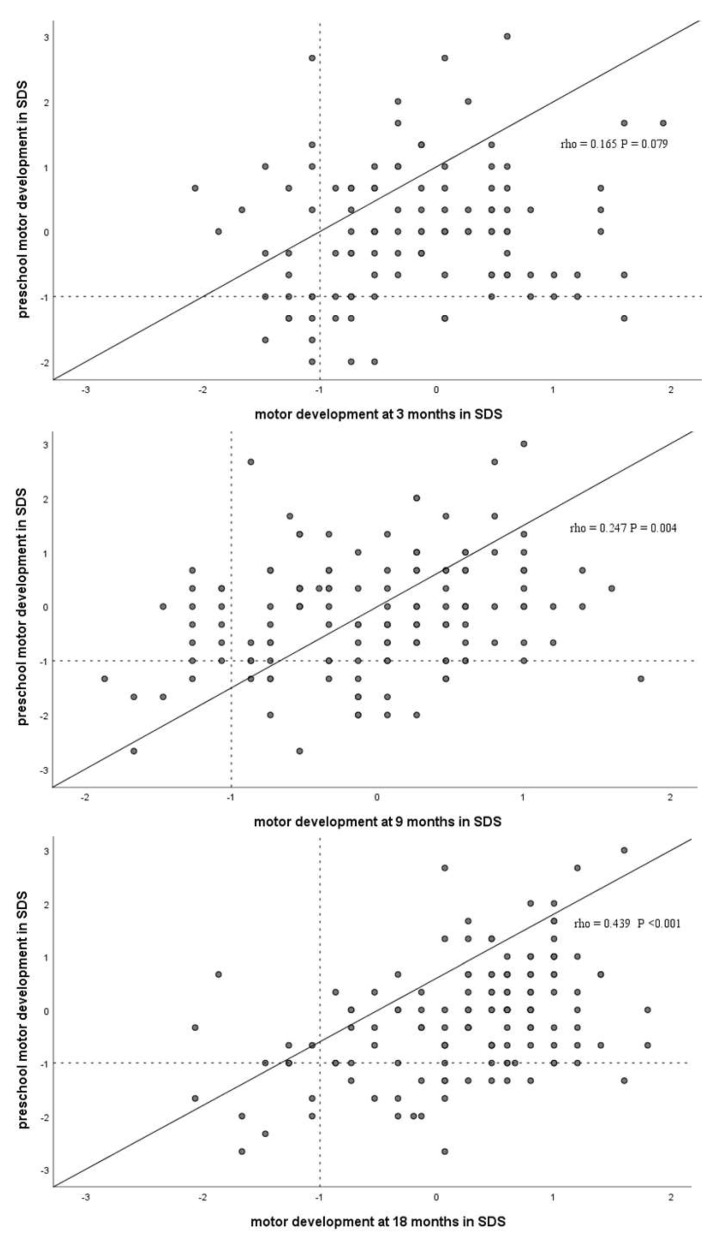
Individual stability between motor developmental measures. Correlations and corresponding *p*-values for motor function are presented for the time intervals 3–42 months, 9–42 months and 18–42 months. Children with unstable development on the different measurements (increase < −1 SD to >−1 SD or decrease > −1 SD to <−1 SD) are in the quadrants at the top left and bottom right.

Intra individual stability of motor developmental outcomes over time was moderate at best. The total motor score at 3 months did not correlate significantly with motor outcomes at 42 months (r (114) = 0.165, *p* = 0.079). The motor score at 9 (r (137) = 0.247, *p* = 0.004) and 18 months (r (140) = 0.439, *p* = <0.001) correlated significantly with motor scores at 42 months (see Figure 2).

**Figure 3:**
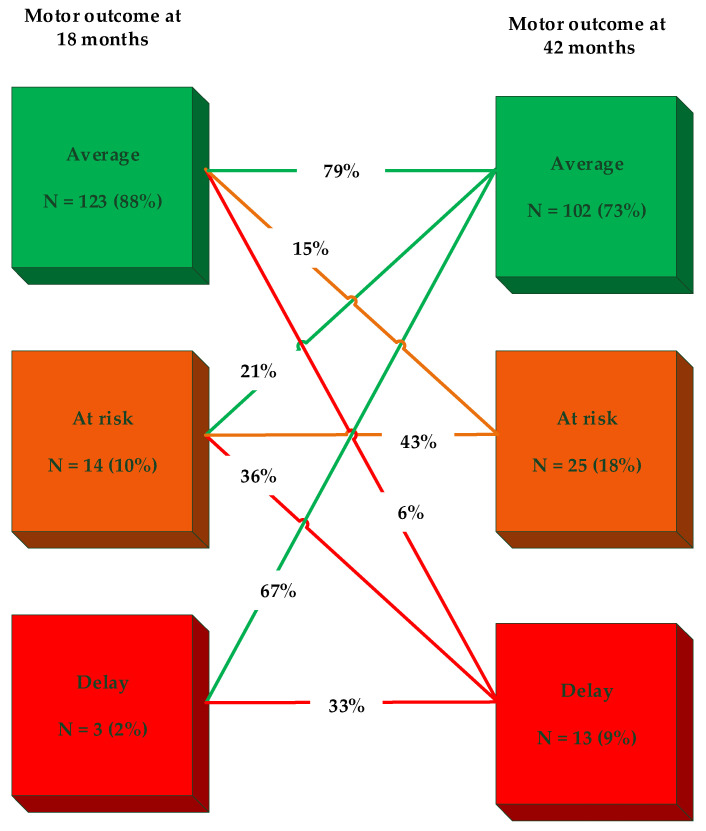
Longitudinal motor development between 18 and 42 months. Scores above the 16th percentile are classified as ‘average’ motor performance. Scores between the 6th and 16th percentile are classified ‘at risk’ for motor difficulties. Scores at or below the 5th percentile indicate a motor ‘delay’.

Hundred-forty children were seen at the outpatient clinic at 18 and 42 months. Motor outcome of 31 (22.2%) children deteriorated between 18 and 42 months. The other children remained stable (74.3%) or even improved (3.5%). For details about longitudinal motor development, see Table 2 and Figure 3.

### 3.5. Predictors for Motor Outcomes at Preschool Age

Motor development at preschool age is significantly associated with gross, fine and total motor skills at 9 and 18 months, sibling status, gestational age, cardio pulmonary bypass (CPB) time, aortic cross clamp (ACC) time, mechanical ventilation time, secondary sternum closure, total admission time, admission time after the first operation, total admission time on the ward, pre-and postoperative admission time in the Pediatric Intensive Care Unit (PICU), number of CCs (including both diagnostic cardiac catheterization and catheter interventions) and number of cardiac surgeries up to 18 and 42 months. Associations between motor development and selected risk factors are presented in Table A2 and Table A3.

All abovementioned factors were considered possible predictors for the multilevel model and were included in the model. Number of cardiac CCs and cardiac surgeries were corrected for diagnosis. Because multiple studies show that sex and cardiac diagnosis are risk factors for adverse neurological outcomes, these variables were also included in the model, although they did not significantly correlate with the motor development up to 42 months.

The fine and gross motor scores at 9 and 18 months positively predicted motor development up to 42 months. Gestational age, CBP time, ACC time and the number of CCs up to 18 months did negatively predict motor development up to 42 months. The final significant multilevel model for motor development is expressed in Table 3.

## 4. Discussion

This study aimed to investigate the motor performance and determinants for unfavorable motor developmental outcomes of preschool children with different types of CCHD who have undergone heart surgery with use of cardio pulmonary bypass before six months of age.

Even though the mean total motor scores from children with a CCHD did not deviate much from healthy peers, a significant proportion of children scored at risk (>P5≤P16) or even delayed (≤P5) at 42 months on the Movement ABC-II-NL. On the total motor score, manual dexterity, aiming and catching (ball skills), and balance skills, 22–27% of the children scored below the 16th percentile, which is normally expected in 16% of the population. Furthermore, a considerable proportion (22.2%) of children showed a deterioration in motor presentation compared to the last consultation at 18 months. Almost 9% of the preschool children scored ≤P5 at 42 months. This number is four times as high as the number reported previously in the same population [16]. This may be a distorted picture as the Bayley-III may have underestimated the number of infants with a motor delay at 18 months [21]. Nevertheless, our findings are in agreement with results from Brosig et al. [14] who reported that many patients who scored in the average range at 24 months showed deficits at 4 years of age. Meanwhile, our findings deviate from recent findings from Naef et al. [13] and Mussatto et al. [9], who reported improvements with age in cognitive and motor functions in early childhood. In these studies, group averages were described, instead of the number of children with a developmental delay.

The (increasing) number of children with motor deficits might be due to acquired brain injury. This could not be confirmed as MRIs were not performed in the majority of infants. Several studies have reported a high prevalence of prenatal and postnatal preoperative and postoperative abnormal cerebral findings, such as delayed brain maturation and brain injury [7,22] and its association with various patient-specific parameters and procedure-specific parameters, which can affect motor development independently, cumulatively and synergistically [5,7,23]. Brain injury, especially white matter injury, occurs particularly in newborns with a SVP and AAA [24,25,26]. Algra et al. [27] found new postoperative brain injury in 72–78% of AAA patients. CPB time is considered one of the risk factors for postoperative brain injury [28]. This may partly explain our finding that the percentage of children scoring below the 5th percentile was highest in the AAA group as the children with AAA had a significantly longer CPB time than the other diagnosis groups.

The lower scores of children with SVP and AAA may also be explained by the use of Deep Hypothermic Circulatory Arrest (DHCA) or Antegrade Cerebral Perfusion (ACP) during aortic arch reconstructions (in AAA patients) and the Norwood procedure (in SVP patients). Either DHCA or ACP is mandatory during these procedures and is associated with an increased risk of postoperative white matter injury [27]. However, this distinction in surgical techniques was not included in the analyses and should be further investigated in future research.

Although the proportion of children whose motor classification had deteriorated compared to the consultation at 18 months was highest in the AAA group (35%), the deterioration in motor outcome was also seen in the other diagnosis groups. This increasing number could result from the cumulative effects of early brain damage on development. As more functions mature during development, consequences such as cognitive impairment and impaired motor function become apparent after a while. This phenomenon is known as “growing into deficits” [29]. In addition to lower motor scores and lower IQ scores, deficits in executive functions and their relation to brain injury are also frequently reported in children and adolescents with CCHD [15,25,30].

The increasing prevalence of abnormal motor outcomes at school age may be due to more complex motor challenges that partly rely on executive functioning. When motor tasks become more complex, different brain functions are needed simultaneously to perform a task successfully. During the administration of the Movement ABC-II-NL, tasks are offered which must be performed as quickly, accurately or as long as possible. Thus, compared to the previously used Bayley-III, a greater appeal is made to higher cognitive functions such as reaction speed, attention, planning, precision and inhibition. These higher cognitive functions are required for behavioral control and change of habits [31]. Limitations in these functions are often leading to difficulties in daily life and could therefore have affected motor performance as well [32].

Finally, family factors like overprotection and parental anxiety, which were not included in this study, may contribute to the increasing number of children with a motor delay. Although overprotection and parental anxiety is regularly mentioned [5,33], the direct relationship between overprotection and motor development in children with CCHD has, to the best of our knowledge, not been investigated yet. In our experience, parents are often protective and concerned about their child, the underlying heart disease and possible risks of (vigorous) physical activity and thus prevent them from exploring their environment and physical abilities and engaging in physical activity. Parents often report that their child is developing beyond expectations, so their expectations seem to be not as high as for their healthy children. As a result, children with a CCHD may be less challenged to learn new age-appropriate motor skills and push their physical boundaries. These factors may also contribute to the increasing number of children with a motor delay.

Motor development of children with CHD between 0 and 42 months is best predicted by early motor outcomes. Pre- peri and postoperative factors have less predictive value, but gestational age, CPB time, cross clamp time, and number of heart catheterizations up to 18 months at least partly predict motor development at preschool age. This is in agreement with previous studies on risk factors for brain injury and adverse motor development in children with a CCHD [28,34,35,36,37]. Cardiac diagnosis did not significantly predict motor outcome at preschool age, which is in line with a recent study from Brosig et al. [15]. However, in previous studies, including one within the same study population, children with SVP were found to score significantly lower than children with other types of heart defects [12,16]. The difference between the different diagnosis groups may have decreased because sixty percent of the children whose motor development had normalized between 18 and 42 months appeared to have a SVP. This improvement over time might be explained by the definitive completion of the Fontan circulation; the total cavo pulmonary connection (TCPC), that occurred in 21 out of 27 SVP patients between 18 and 42 months, resulting in an improvement in oxygen saturation, followed by improved energy- and activity levels, which allows more opportunities to practice and improve motor skills.

Lastly, motor development up to preschool age in this group was not predicted by sex, Apgar scores, type of delivery, antenatal diagnosis, BAS procedures, need for mechanical ventilation support prior to neonatal surgery, postoperative LCOS and need for ECLS, which is in contrast to findings in previous studies. The conclusion of a recent systematic review of Huisenga et al. on risk factors for adverse motor developmental outcomes in infants with a CCHD already showed that perioperative factors are inconsistently associated with outcomes. These differences may be partly explained by differences between hospitals in treatment strategies, definitions and cut-off points in classifying risk factors, but the interplay of different risk factors for brain injury and adverse long-term outcomes appears to be multifactorial and is still not fully understood [5,23].

There are several limitations to the current study. All children had been previously evaluated one to three times in our developmental follow-up. After these follow-up moments, parents are often given advice on how they can support the development of their child as optimally as possible, which probably leads to more favorable motor outcomes. In addition, children with significant motor disabilities are less likely to have completed the follow-up assessment at 42 months. Furthermore, in 13 children, the motor assessment could not be completed because they were not cooperative enough to reliably test their motor development. Lastly, of the eligible patients, six were diagnosed with cerebral palsy. For four of them, no assessment was available. Therefore, mean motor scores may be overestimated and the prevalence of children with motor delays underestimated.

The results are based on a single-center study, not all eligible patients participated in clinical follow-up and not all patients consented to the use of the clinical data. Lastly, children with underlying genetic abnormalities and preterm infants have been excluded. Our findings are therefore not generalizable to the CCHD population as a whole.

Large prospective studies in unselected populations of infants with a CCHD are scarce. To the best of our knowledge, this is one of a few studies in which such a specific group of term-born infants without underlying genetic anomalies was followed longitudinally, which can be seen as a strength of our study. The longitudinal development of children with a CHD has been described previously, but to our knowledge, early motor outcome as a predictor of motor activity at preschool age has rarely been studied [9,13]. Based on our results concerning longitudinal motor trajectories of term born children with a CCHD without a underlying genetic defect, a cautious prognosis can be made with regard to the expected development at 42 months.

Based on the current study, it is recommended that children with a CCHD should be regularly monitored for their (motor) development. Especially children with younger gestational age, longer CPB time, longer cross-clamp time and those with a larger number of CCs up to 18 months are at increased risk of developing motor problems at preschool age. Early motor development appears to be an independent predictor of exercise capacity, physical activity, participation in daily living, and the frequency of sports activities in childhood [38]. Nevertheless, motor developmental stability is moderate which makes the prediction of future motor outcome difficult. This emphasizes the need for ongoing systematic screening on the emergence of developmental problems for all children with CCHD to protect their current and future cardiovascular health. Consideration should be given to offering early interventions, particularly when motor outcomes are less favorable at 9 and 18 months of age.

It is still unknown whether preschool motor outcomes will predict motor outcomes at school age and beyond. Additional research into the prevalence of developmental problems in motor competence, exercise capacity, physical activity and (sports) participation as well as the influence of environmental factors such as parenting style in school-age children with CCHD is recommended. In future studies, more attention should be paid to the role of ‘physical literacy’. Physical literacy is the development of fundamental movement and sports skills that allow children to move confidently and competently in a wide range of physical activities and sports situations. Higher levels of physical literacy in healthy children are associated with higher levels of physical activity [39]. As motivation, confidence, physical competence, understanding and knowledge are essential to remain physically active [40], more attention should be paid to the role of such factors in addition to the contribution of medical factors.

Although most infants with CCHD display an average total motor development at school age, the number of infants with a delayed motor development is increased and increases with age in all CCHD subtypes. The percentage of children whose motor development improved between 18 and 42 months was highest in the SVP group; the number of children whose development deteriorated was highest in the AAA group. In particular, children with younger gestational age, longer CPB time, longer cross-clamp time, as well as those with a larger number of CCs up to 18 months and less favorable motor outcomes at 9 and 18 months of age, were at increased risk of developing motor problems at school age. This information could contribute to the further development and implementation of early intervention strategies, long-term developmental follow-up pathways, and more individualized developmental supportive care.

## Figures and Tables

**Table 1:** Patient characteristics.

	Total *n* = 147	TGA *n* = 72	SVP *n* = 27	TOF *n* = 25	AAA *n* = 15	Others *n* = 8
**Male**	92 (63)	50 (69)	13 (48)	14 (56)	11 (73)	4 (50)
**Birth weight**, *grams* *	3450 ± 560	3559 ± 516	3345 ± 474	3154 ± 595	3547 ± 762	3582 ± 364
**Gestational age**, *weeks* *	39.5 ± 1.4	39.6 ± 1.4	39.2 ± 1.2	39.0 ± 1.5	39.9 ± 1.3	40.1 ± 0.7
**Apgar score 5 min**	9 (8–10)	8 (8–9)	8.5 (7.5–9)	9 (8.5–9)	10 (10–10)	9 (8.5–9.5)
	8.8 ± 1.0					
**Type of delivery**						
Spontaneous vaginal	62 (42)	35 (49)	6 (22)	11 (44)	5 (33)	5 (63)
Induced vaginal	36 (25)	18 (25)	11 (41)	6 (24)	1 (7)	0 (0)
Cesarean Section	33 (20)	15 (21)	7 (26)	3 (12)	6 (40)	2 (25)
Unknown	16 (11)	4 (5)	3 (11)	5 (20)	3 (20)	1 (12)
**Prenatal diagnosis**	98 (67)	48 (67)	24 (88)	16 (64)	9 (60)	1(12)
**Balloon Atrioseptostomy (BAS)**	48 (33)	44 (61)	3(11)	0 (0)	1 (7)	0 (0)
**Age at surgery** *(days)*	10 (7–23)	9 (7–10)	7 (5.5–8.0)	65 (28–94)	9.5 (8–11)	68 (35–71)
	25.7 ± 36.0					
**Intubated pre-operative**	75 (51)	51 (71)	10 (37)	2 (8)	6 (40)	6 (75)
**Total mechanical ventilation time (days)**	4.3 (2.2–8.0)	4.3 (3.0–7.4)	7.9 (3.8–15.4)	0.9 (0.2–3.0)	4.5 (3.0–10.5)	3.8 (2.0–16.3)
	6.8 ± 10.2					
**Deep Hypothermic Cardiac Arrest (DHCA)**	36 (24)	4 (6)	17 (63)	0	15 (100)	0
**Antegrade Cerebral Perfusion (ACP)**	30 (20)	4 (6)	15 (56)	0	11 (73)	0
**Aortic cross clamp (ACC) time** *(minutes)*	79 (61–97)	94 (79–105)	74 (60–88)	65 (28–71)	88 (80–96)	76 (61–102)
	78.9 ± 39.0					
Cardio pulmonary bypass (CPB) time * *(minutes)*	134 ± 50.0	145 ± 44.1	133 ± 59.5	94 ± 25.3	165 ± 45.2	107 ± 53.1
**Low Cardiac Output Syndrome (LCOS) postoperative**	30 (20)	16 (22.2)	13 (48.1)	0	1 (6.7)	0
**Need for Extra Corporeal Life Support (ECLS)**	2 (1)	0	2 (7.4)	0	0	0
**Total length of hospital stay** *(days)*	19 (15–27)	18 (16–20)	118 (36–127)	27 (13–37)	19 (17–21)	24 (18–37)
	29.5 ± 32.6					
**Postoperative hospital stay** *(days)*	11 (8–16.5)	9 (7–12)	113 (31–120)	12 (8–22)	10 (9–10)	15 (13–33)
	22.3 ± 32.9					
**Total Pediatric Intensive Care Unit (PICU) stay** *(days)*	9 (6–13)	10 (7–13)	16 (14–26)	7 (4–11)	6 (4–8)	8 (7–20)
	12.5 ± 15.1					
**PICU stay** pre-operative *(days)*	3 (1–5)	4 (2–7)	5 (2–6)	1 (0–4)	1 (0–2)	1 (1–2)
	3.4 ± 3.3					
**PICU stay** postoperative *(days)*	5 (3–8)	5 (3–6)	13 (8–24)	6 (3–9)	5 (4–6)	7 (6–19)
	9.0 ± 14.5					
**Total stay on ward** *(days)*	10 (7–16)	8 (5–11)	89 (23–103)	18 (9–24)	13 (13–13)	18 (11–19)
	17.1 ± 24.6					
**Stay on ward** pre-operative *(days)*	3 (0–7)	3 (0–5)	0 (0–2)	9 (6–13)	9 (8–9)	0 (0–4)
	3.78 ± 4.0					
**Stay on ward** postoperative *(days)*	6 (4–9)	5 (3–6)	89 (21–99)	6 (4–10)	5 (4–5)	11 (7–15)
	13.3 ± 25					
**Number of cardiac surgeries**	1 (1–2)	1(1–1)	2(2–2)	1 (1–2)	1(1–1)	1 (1–2.5)
**≤18 months**	1.33 ± 0.55	1.13 ± 0.33	1.96 ± 0.34	1.28 ± 0.54	1.27 ± 0.59	1.38 ± 1.06
1 operation	103 (70)	63 (88)	2 (7)	19 (76)	12 (80)	7 (88)
2 operations	40 (27)	9 (12)	24 (89)	5 (20)	2 (13)	0 (0)
3 operations	3 (2)	0 (0)	1 (4)	1 (4)	1 (7)	0 (0)
4 operations	1(1)	0 (0)	0 (0)	0 (0)	0 (0)	1 (12)
**Number of cardiac catheterization’s (CCs)**	1 (0–1)	1 (0–1)	2 (1.5–3)	0 (0–2)	0.5 (0–1)	1 (0.5–2)
**≤18 months**	0.95 ± 1.15	0.67 ± 0.53	2.41 ± 1.45	0.56 ± 1.16	0.53 ± 0.74	0.63 ± 1.06
**Number of cardiac surgeries**	1 (1–2)	1(1–1)	3(3–3)	1 (1–2)	1.5(1–2)	1 (1–2.5)
**≤42 months**	1.5 ± 0.80	1.13 ± 0.33	2.78 ± 0.43	1.28 ± 0.54	1.47 ± 0.83	1.38 ± 1.06
1 operation	99 (67)	63 (88)	0	19 (76)	10 (67)	7 (88)
2 operations	24 (16)	9 (12)	6 (22.2)	5 (20)	4 (27)	0 (0)
3 operations	22 (15)	0 (0)	21(77.8)	1 (4)	0 (0)	0 (0)
4 operations	2 (1)	0 (0)	0	0 (0)	1 (7)	1 (12)
**Number of CCs**	1 (0–1)	1 (0–1)	4 (2.5–4)	0.5 (0–2)	0.5 (0–1)	1 (0.5–2)
**≤42 months**	1.18 ± 1.47	0.69 ± 0.57	3.37 ± 1.71	0.68 ± 1.25	0.67 ± 0.98	0.7 ± 1.04

Data are presented as mean ± standard deviation (* normally distributed) or as median with 25th/75th centiles (not normally distributed) or as number with percentage. Described data are pre- peri- and postoperative data from the first neonatal cardiac surgery using the CPB. LCOS is defined as lactate > 4 and pH < 7.30 on at least two consecutive measurements.

**Table 2:** Movement Assessment Battery for children–II-NL: Motor outcomes of preschoolers with a critical congenital heart defect (CCHD).

Movement ABC-II-NL Percentile Scores	Total	TGA	SVP	TOF	AAA	Others	*p* Value
**42 months**	*n* = 147	*n* = 72	*n* = 27	*n* = 25	*n* = 15	*n* = 8	
**Total motor score**	9.5 ± 3.14	10.1 ± 3.18	8.5 ± 2.50	10.0 ± 3.39	7.9 ± 3.31	9.0 ± 2.27	0.041 *
>P16	107 (72.8)	53 (73.6)	19 (70.4)	20 (80.0)	9 (60.0)	6 (75.0)	
>P5≤P16	27 (18.4)	16 (22.2)	5 (18.5)	2 (8.0)	3 (20.0)	1 (12.5)	
≤P5	13 (8.8)	3 (4.2)	3 (11.1)	3 (12.0)	3 (20.0)	1 (12.5)	
**Domain standard scores**							
Manual dexterity	9.7 ± 3.09	10.1 ± 3.06	8.9 ± 3.06	10.4 ± 2.63	8.1 ± 3.75	9.1 ± 2.23	0.053
>P16	113 (76.9)	57 (79.2)	19 (70.4)	21 (84.0)	10 (66.7)	6 (75.0)	
>P5≤P16	25 (17)	13 (18.1)	5 (18.5)	4 (16.0)	1 (6.7)	2 (25.0)	
≤P5	9 (6.1)	2 (2.8)	3 (11.1)	0 (0.0)	4 (26.7)	0 (0.0)	
Aiming and catching	9.4 ± 2.68	9.7 ± 2.52	9.2 ± 2.59	9.0 ± 3.55	9.1 ± 2.17	9.4 ± 2.56	0.73
>P16	115 (78.2)	58 (80.6)	21 (77.8)	16 (64.0)	13 (86.7)	7 (87.5)	
>P5≤P16	19 (12.9)	10 (13.9)	4 (14.8)	4 (16.0)	1 (6.7)	0 (0.0)	
≤P5	13 (8.8)	4 (5.6)	2 (7.4)	5 (20.0)	1 (6.7)	1 (12.5)	
Balance	9.7 ± 3.30	10.2 ± 3.6	8.9 ± 2.09	10.0 ± 3.66	8.5 ± 3.11	9.3 ± 2.25	0.39
>P16	113 (76.9)	56 (77.8)	2 (81.5)	19 (76.0)	9 (60.0)	7 (87.5)	
>P5≤P16	26 (17.7)	13 (18.1)	3 (11.1)	5 (20.0)	4 (26.7)	1 (12.5)	
≤P5	8 (5.4)	3 (4.2)	2 (7.4)	1 (4.0)	2 (13.3)	0 (0.0)	
**42 months vs. 18 months **	*n* = 140	*n* = 69	*n* = 25	*n* = 24	*n* = 14	*n* = 8	
**Motor score deteriorated**	31 (22.1)	15 (21.7)	5 (20.0)	5 (13.5)	5 (35.7)	1 (12.5)	
**Motor score improved**	5 (3.6)	2 (2.9)	3 (12)	0 (.0)	0 (0.0)	0 (0.0)	
**Motor score equal**	104 (74.3)	52 (75.4)	17 (68)	19 (2.2)	9(64.3)	7 (87.5)	

Data are presented as mean (+/− standard deviation) or as number with percentage. * Statistically significant difference *p*-value < 0.05. Abbreviations: CCHD: critical congenital heart disease; SVP: single ventricle physiology; TGA: transposition of great arteries; AAA: aortic arch anomaly; TOF: tetralogy of Fallot; FM SS: Fine Motor Scaled Score; GM SS: Gross Motor Scaled Score.

**Table 3:** Final multilevel model predicting total motor development.

Model	Estimate (S.E)	Level 2 Variance	Level 1 Variance	Log Likelihood (χ^2^)	*p*-Value Model
Empty model intercept	−0.047 (0.051)	0.224 (0.046)	0.570 (0.041)	1354.943	
Final model intercept	−4.671 (1.003)				
+Gestational age	0.040 (0.025)			1326.902	<0.001
+CPB time (min)	−0.0432 (0.080)			1301.536	<0.001
+Cross clamp time (hours)	−0.009 (0.108)			1232.105	<0.001
+Number CCs up to 18 months	−0.032 (0.036)			1223.814	0.004
+Bayley-III-NL GMSS 9 months	0.052 (0.013)			1100.542	<0.001
+Bayley-III-NL FMSS 9 months	0.076 (0.022)			1089.136	<0.001
+Bayley-III-NL GMSS 18 months	0.085 (0.012)			1007.887	<0.001
+Bayley-III-NL FMSS 18 months	0.097 (0.019)	0.000 (0.000)	0.470 (0.031)	978.763	<0.001

Abbreviations: Bayley III-NL = Bayley Scales of Infant and Toddler Development, Dutch edition; GMSS = Gross Motor Scaled Score; FMSS = Fine Motor Scaled Score.

## Data Availability

Data are available from the corresponding author.

## Appendix A

**Table A1:** Overview of first surgical procedure.

	Total	TGA	SVP	TOF	AAA	Others
	*n* = 147	*n* = 72	*n* = 27	*n* = 25	*n* = 15	*n* = 8
**Type of cardiac surgery**						
Arterial switch	39 (26.5)	39 (54.2)	-	-	-	-
Arterial switch with aortic arch repair	1 (0.7)	1 (1.4)	-	-	-	-
Arterial switch with VSD closure	20 (13.6)	20 (27.8)	-	-	-	-
Arterial switch with aortic arch repair and VSD closure	4 (2.7)	4 (5.6)	-	-	-	-
Norwood	21(14.3)	-	21 (77.8)	-	-	-
AP-shunt	13 (8.8)	6 (8.3)	3 (11.1)	4 (16.0)	-	-
PCPC	1 (0.7)	-	1 (3.7)	-	-	-
ToF correction	21 (14.3)	-	-	21 (84.0)	-	-
TAPVC correction	4 (2.7)	-	-	-	-	4 (50.0)
Aortic arch repair	6 (4.1)	-	-	-	6 (40.0)	-
Aortic arch repair with VSD closure	7 (4.8)	-	-	-	7 (46.7)	-
Aortic arch repair with Ross Konno procedure	1 (0.7)	-	-	-	1 (6.7)	-
VSD closure	2 (1.4)	-	-	-	-	2 (25.0)
Ross Konno procedure	1 (0.7)	-	-	-	-	1 (12.5)
DKS procedure and BT shunt	2 (1.4)	-	2 (7.4)	-	-	-
Yasui operation	1 (0.7)	-	-	-	1 (6.7)	
Brom Aortoplasty	1 (0.7)	-	-	-	-	1 (12.5)
Nikaidoh procedure	1 (0.7)	1 (1.4)	-	-	-	-
Other	1 (0.7)	1 (1.4)	-	-	-	-

Data are presented as number with percentage. Abbreviations: SVP: single ventricle physiology; TGA: transposition of great arteries; AAA: aortic arch anomaly; TOF: tetralogy of Fallot AP-shunt: aorto pulmonary shunt; PCPC: partial cavo pulmonary circulation; TAPVC: total anomalous pulmonary venous return; VSD: ventricular septal defect; DKS: Damus–Kaye–Stansel; BT; Blalock–Taussig.

## Appendix B

**Table A2:** Correlations between motor outcome and selected risk-factors.

N = 147	M-ABC-II Total Motor Development		M-ABC-II Manual Dexterity		M-ABC-II Aiming and Catching		M-ABC-II Ball Skills	
	Spearman’s Rho	*p*-Value	Spearman’s Rho	*p*-Value	Spearman’s Rho	*p*-Value	Spearman’s Rho	*p*-Value
Age at surgery *(days)*	0.012	0.89	0.075	0.36	−0.053	0.52	−0.009	0.92
CPB time *(minutes)*	−0.129	0.12	−0.186	**0.03**	−0.125	0.14	−0.075	0.37
ACC time *(minutes)*	−0.094	0.27	−0.115	0.18	−0.170	**0.045**	−0.041	0.63
Total mechanical ventilation time *(days)*	−0.154	0.06	−0.212	**0.01**	−0.141	0.1	0.002	0.98
Gestational age (weeks)	0.170	**0.04**	0.101	0.23	0.257	** <0.01 **	0.098	0.24
Apgar-1	−0.054	0.55	−0.063	0.48	−0.008	0.09	−0.101	0.26
Apgar-5	0.007	0.94	−0.020	0.82	0.044	0.63	−0.039	0.67
Apgar-10	0.038	0.78	−0.049	0.71	0.102	0.45	0.030	0.82
Birthweight (grams)	0.066	0.43	0.036	0.67	0.056	0.50	0.101	0.23
PICU stay pre-operative *(days)*	0.066	0.43	−0.038	0.65	0.047	0.57	0.177	**0.03**
Stay on ward pre-operative *(days)*	0.011	0.89	0.049	0.55	0.019	0.82	0.006	0.94
Pre-operative hospital stay *(days)*	−0.009	0.92	0.017	0.84	−0.016	0.84	0.072	0.39
PICU stay postoperative *(days)*	−0.158	0.06	−0.199	**0.02**	−0.141	0.09	−0.026	0.75
Stay on ward postoperative *(days)*	−0.099	0.23	−0.152	0.07	−0.085	0.31	−0.007	0.94
Postoperative hospital stay *(days)*	−0.119	0.15	−0.188	**0.02**	−0.096	0.25	0.007	0.93
Total Paediatric Intensive Care Unit (PICU) stay *(days)*	−0.090	0.28	−0.155	0.06	−0.119	0.15	0.061	0.46
Total stay on ward *(days)*	−0.170	**0.04**	−0.172	**0.04**	−0.11	0.18	−0.074	0.38
Total length of hospital stay *(days)*	−0.156	0.06	−0.201	**0.02**	−0.130	0.12	0.004	0.96
Number of CCs < 18 months	−0.151	0.07	−0.216	** <0.01 **	−0.053	0.53	−0.076	0.36
Number of cardiac surgeries < 18 months	−0.174	**0.04**	−0.151	0.07	−0.150	0.07	−0.053	0.53
Number of CCs < 42 months	−0.069	0.14	−0.203	**0.01**	−0.041	0.62	−0.069	0.41
Number of cardiac surgeries < 42 months	−0.207	**0.01**	−0.180	**0.03**	−0.157	0.06	−0.083	0.32
Bayley-III-total motor score 3 months	0.017	0.08	0.034	0.72	0.249	** <0.01 **	0.158	0.09
Bayley-III-FMSS 3 months	0.010	0.28	0.011	0.91	0.196	**0.04**	0.104	0.27
Bayley-III-GMSS 3 months	0.012	0.19	0.030	0.76	0.189	**0.04**	0.101	0.29
Bayley-III-total motor score 9 months	0.247	** <0.01 **	0.230	**0.01**	0.188	**0.03**	0.184	**0.03**
Bayley-III-FMSS 9 months	0.198	**0.02**	0.205	**0.02**	0.183	**0.03**	0.091	0.29
Bayley-III-GMSS 9 months	0.218	**0.01**	0.188	**0.03**	0.154	0.073	0.192	**0.03**
Bayley-III-total motor score 18 months	0.439	** <0.001 **	0.411	** <0.001 **	0.222	** <0.01 **	0.383	** <0.001 **
Bayley-III-FMSS 18 months	0.366	** <0.001 **	0.363	** <0.001 **	0.218	**0.01**	0.251	** <0.01 **
Bayley-III-GMSS 18 months	0.358	** <0.001 **	0.331	** <0.001 **	0.161	0.056	0.346	** <0.001 **

Bold numbers represent significant linear relation indicated by Spearman’s Rho (*p* < 0.05), *n* = 147. Abbreviations: ACC Time: Aortic Cross Clamp Time; CPB time: Cardio Pulmonary Bypass Time; CCs: Cardiac Catheterizations; PICU: Pediatric Intensive Care Unit; Bayley-III Bayley Scales of Infant and Toddler Development–third edition; FMSS: Fine Motor Scaled Score; GMSS: Gross Motor Scaled Score. M-ABC-II: Movement Assessment Battery for Children—second edition

## Appendix C

**Table A3:** Correlations between motor classification and selected risk factors.

*n* = 147	M-ABC-II Total Motor Development		M-ABC-II Manual Dexterity		M-ABC-II Aiming and Catching		M-ABC-II Ball Skills	
	Chi-Square (df)	*p*-Value (2-Sided)	Chi-Square (df)	*p*-Value (2-Sided)	Chi-Square (df)	*p*-Value (2-Sided)	Chi-Square (df)	*p*-Value (2-Sided)
Gender		0.47 ^(b)^		0.12 ^(b)^		0.68 ^(b)^		0.20 ^(b)^
Cardiac diagnosis		0.29 ^(b)^		0.08 ^(b)^		0.63 ^(b)^		0.53 ^(b)^
Antenatal diagnosis (yes/no)		0.88 ^(b)^		0.74 ^(b)^		0.83 ^(b)^		0.53 ^(b)^
Type of delivery		0.89 ^(b)^		0.58 ^(b)^		0.57 ^(b)^		0.31 ^(b)^
BAS (yes/no)		0.41 ^(b)^		0.38 ^(b)^		0.33 ^(b)^		0.80 ^(b)^
Secondary sternum closure (yes/no)		0.37 ^(b)^		**0.02** ^(b)^		0.50 ^(b)^		0.06 ^(b)^
Need for mechanical ventilation support pre-operative (yes/no)	1.568 (2)	0.46 ^(a)^		0.19 ^(b)^	4.467 (2)	0.12 ^(a)^		0.38 ^(b)^
Low Cardiac Output Syndrome (LCOS) postoperative (yes/no)		0.07 ^(b)^		0.30 ^(b)^		0.11 ^(b)^		0.21 ^(b)^
ECLS (yes/no)		0.47 ^(b)^		0.41 ^(b)^		0.19 ^(b)^		1.00 ^(b)^

Bold numbers represent significant relations indicated by chi square ^(a)^ and fishers exact ^(b)^; Abbreviations: M-ABC-II: Movement Assessment Battery for Children-second edition; BAS: Balloon Atrio Septosomy; LCOS: Low Cardiac Output Syndrome; ECLS: Extra Corporeal Life Support.
